# Anti-nephrin autoantibodies in podocytopathies: pathogenicity, assay discordance, and translation

**DOI:** 10.3389/fimmu.2026.2000932

**Published:** 2026-10-26

**Authors:** Peidong Feng, Xiaoyong Yu

**Affiliations:** 1 Shaanxi University of Chinese Medicine, Xianyang, Shaanxi, China; 2 Department of Nephrology, Shaanxi Provincial Hospital of Traditional Chinese Medicine, Xi’an, Shaanxi, China

**Keywords:** autoantibody, focal segmental glomerulosclerosis, immunoassay, minimal change disease, nephrin, nephrotic syndrome

## Abstract

Experimental targeting of nephrin causes proteinuria and podocyte injury, and human studies identify anti-nephrin autoantibodies in a subset of podocytopathies. Serum and tissue recognition, associations with disease activity, and transfer of patient total immunoglobulin G support an autoimmune endotype. The pathogenic contribution of the naturally occurring nephrin-specific fraction has not been fully resolved. This narrative review examines target recognition, pathogenic effects, clinical validity, and the benefit of acting on a test result as separate questions. We focus on adult minimal change disease and primary focal segmental glomerulosclerosis, with childhood nephrotic syndrome and transplant recurrence providing complementary evidence. Experimental studies explain how nephrin targeting alters slit diaphragm organization and signaling. Comparisons of original studies identify glycan and tag interference, platform-dependent detection, and differences in treatment exposure and antibody class. Low detection with human-cell antigens also supports considering genuine variation in the frequency of this endotype. Observational studies show serologic changes with disease activity and signals preceding relapse or transplant recurrence. These findings motivate investigational applications in disease characterization, monitoring, and risk assessment. Assays remain unstandardized, and autoantibody-guided management has not been shown to improve outcomes over standard care. Prospective studies and decision-focused trials should determine when these signals add useful information and improve treatment.

## 1 Introduction: antigen-specific autoimmunity in podocytopathies

Minimal change disease (MCD) and focal segmental glomerulosclerosis (FSGS) describe kidney lesions. Nephrotic syndrome describes a clinical state, and steroid sensitivity describes a treatment response. An antigen-specific autoimmune mechanism adds a different layer of information. These layers overlap, but their boundaries matter when a new blood test is proposed for diagnosis or treatment selection.

Watts and colleagues identified circulating autoantibodies against nephrin in a subset of patients with MCD. They linked serum reactivity to nephrin recognition, disease activity, and punctate immunoglobulin G (IgG) in kidney biopsies (1). A larger study by Hengel and colleagues extended the finding across adult MCD, primary FSGS, and childhood idiopathic nephrotic syndrome. Its immunization experiments also showed that an immune response against nephrin could produce nephrotic disease (2). These findings support a nephrin-directed autoimmune endotype within diseases previously grouped under an idiopathic label, although its frequency and causal boundaries are still being defined.

The clinical question has become more precise: which autoantibody responses damage podocytes, and which measurements can guide care? A convincing pathogenic experiment does not determine the sensitivity of a clinical assay. A reproducible serum result does not by itself establish that changing treatment on that result benefits the patient. We organize this review around four questions: whether a test detects nephrin-specific binding, whether that response causes injury, whether it identifies or predicts a clinically relevant state, and whether acting on it improves outcomes.

Throughout this review, we use ‘anti-nephrin autoantibodies’ for naturally occurring responses to self-nephrin in patients and ‘anti-nephrin antibodies’ for experimentally raised or commercially produced reagents. Graft-induced alloantibodies and responses elicited by active immunization are identified separately. Clinical terms such as ‘autoantibody-positive’ and ‘autoantibody-guided’ refer to the human anti-nephrin autoantibody response.

### 1.1 Search strategy and evidence selection

This targeted narrative review focuses on adult MCD and primary FSGS, with pediatric and transplant studies providing complementary evidence. PubMed was searched on September 16, 2026, through the NCBI E-utilities interface using three title/abstract queries, without explicit language or publication-date filters.

Reference lists and accessible original reports were also checked, and the relevant ClinicalTrials.gov entry was consulted. We used the following queries:

Main query: (nephrin[Title/Abstract] OR NPHS1[Title/Abstract]) AND (autoantibod*[Title/Abstract] OR autoimmun*[Title/Abstract] OR “anti-nephrin”[Title/Abstract] OR antinephrin[Title/Abstract]).

Assay query: nephrin[Title/Abstract] AND (antibody[Title/Abstract] OR antibodies[Title/Abstract]) AND (assay*[Title/Abstract] OR ELISA[Title/Abstract] OR immunoprecipitation[Title/Abstract] OR glycosylation[Title/Abstract] OR “alpha-Gal”[Title/Abstract]).

Mechanism query: nephrin[Title/Abstract] AND (antibody[Title/Abstract] OR antibodies[Title/Abstract] OR Nck[Title/Abstract]) AND (proteinuria[Title/Abstract] OR phosphorylation[Title/Abstract] OR polymerization[Title/Abstract] OR slit[Title/Abstract]).

During revision, eligibility criteria were applied retrospectively to the saved records for the targeted assay comparison in Section 4. Eligible reports provided original human serum or plasma anti-nephrin autoantibody data in acquired podocytopathies and relevant comparison groups. They included cohort detection results, direct assay or threshold comparisons, analytical validation, or findings on specificity, interference, or epitope characterization. Primary-data letters were eligible, and positive, negative, weak, and discordant findings were assessed using the same criteria. This comparison excluded reports without original human circulating-assay evidence, tissue-only studies, and reports outside the target population. Selected clinical cases and mechanistic studies were retained as context when they lacked a cohort detection analysis or direct assay comparison. Eligibility remained indeterminate when the available source material was insufficient.

The queries returned 171, 58, and 172 records, respectively, yielding 283 unique PMIDs after deduplication; two additional unique seed records produced the saved corpus of 285 records. The query counts reflect PubMed content on September 16, 2026. Because the queries have no date limit, running them later may yield different counts as the database is updated. Reassessment identified 24 eligible reports, 253 records outside the targeted comparison, and eight indeterminate records. These counts refer to reports, not independent patient cohorts. **Table 1** presents five illustrative studies showing differences in antigen preparation, assay readout, thresholds, or sampling context. Extraction distinguished reports, platforms, clinical groups, and sampling time points and recorded denominators, disease activity, treatment exposure, antigen characteristics, positivity rules, results, and source limitations. **Supplementary Methods S1** and **Supplementary Table S1** provide the search strategy and screening and extraction records.

**Table 1:** Sources of anti-nephrin assay discordance.

Study and samples	Antigen and platform	Treatment exposure at sampling	Positivity and calibration	Main result and interpretation
Watts et al, 2022 (1) 62 patients with active, biopsy-proven MCD.	HEK293-F human nephrin, C-terminal 6×His; plate ELISA.	In the NEPTUNE cohort, 97% had started therapy before collection of the earliest available serum sample.	187 U/mL (highest of 30 healthy controls); positive patient serum calibrator.	18/62 positive; active sera precipitated native glomerular nephrin; 11 paired positives became negative in remission.
Hengel et al, 2024 (2) Adult MCD/FSGS and childhood INS.	HEK293-6E human nephrin, C-terminal 8×His + Twin-Strep; IP-WB and IP-ELISA.	Immunosuppression at collection was reported in 37/93 adults with MCD, 32/65 with primary FSGS, and 119/182 children with INS. Adult data were missing for 12 MCD and 9 FSGS patients.	Blinded specific-band assessment with controls; recombinant-nephrin standard quantified recovered nephrin.	46/105 adult MCD positive; 69% in untreated active MCD. Conventional ELISA did not separate IP-positive and negative sera: platform matters.
Liu et al, 2025 (18) Disease/control cohorts; additional MCD 21/FSGS 21.	NS0/HEK293 His-tagged and HEK293 FLAG-tagged human nephrin; plate ELISA, IP-WB, on-bead ELISA.	Sampling relative to immunosuppression was not reported in the accessible main text.	Human-cell ELISA: highest healthy value + group SD; weak IP-WB classification depended on the band threshold.	Tag switching resolved selected false positives. Two FSGS samples remained strongly positive; about half of additional MCD samples had weak IP-WB signals missed by on-bead ELISA.
Pecoraro et al, 2026 (22) 39 SSNS (9 active), 26 SRNS; transplant cohorts: 34 patients with posttransplant recurrence and 4 without recurrence; 14 membranous nephropathy and 20 healthy controls.	NS0 human nephrin Gln23-Thr1029 versus HEK293 Met1-Ser1055; ELISA, IP-WB and α-Gal experiments.	30/39 SSNS samples were obtained during steroid-induced remission. Of the 34 patients in the recurrence group, 7 were sampled during recurrence before plasmapheresis/rituximab; 6/7 were receiving tacrolimus. The remaining 27 were sampled before transplantation (n = 5) or posttransplant (n = 22).	NS0: 180 AU/mL; HEK293: OD450 0.150. See cutoff derivation below.	NS0: 11/20 healthy positive; α-Gal absorption/removal reduced reactivity. HEK293: active SSNS 1/9 (all SSNS 1/39), SRNS 1/26, recurrence-history cohort 1/34, healthy 1/20. Low detection persisted.
Hengel et al, 2026 (16) 17 baseline adult sera compared across readouts.	Twin-Strep-tagged human nephrin; IP-WB versus quantitative IP-ELISA.	Sampling: 17 baseline samples before steroid initiation; 22 after 8 weeks of high-dose steroids (39 samples from 26 adults).	IP-WB: nephrin band. IP-ELISA: 25 RU/mL with recombinant-nephrin calibration.	Same 17 samples: 12 IP-WB-positive; 9 above the quantitative cutoff. Other 3: 5, 10 and 24 RU/mL. Classification differed by readout and cutoff in the same samples.

These selected studies illustrate antigen interference, platform differences, and positivity thresholds. Cohorts differ in patient selection, disease activity, treatment exposure, antigen preparation, and positivity thresholds. Detection proportions and diagnostic performance therefore cannot be compared directly or used to rank assays. Results use the denominators reported in each study and are not pooled. No immunosuppression at collection does not exclude prior treatment. Units and thresholds are assay-specific. Pecoraro cutoffs were mean + 2 SD: α-Gal-negative healthy controls for NS0; healthy controls after excluding one positive sample for HEK293. AU/RU, arbitrary/relative units; ELISA, enzyme-linked immunosorbent assay; FSGS, focal segmental glomerulosclerosis; HEK293, human embryonic kidney 293; INS, idiopathic nephrotic syndrome; IP-WB, immunoprecipitation-Western blotting; MCD, minimal change disease; OD450, optical density at 450 nm; SD, standard deviation; SRNS/SSNS, steroid-resistant/sensitive nephrotic syndrome.

During revision, PF re-screened the saved records and re-extracted the evidence. XY subsequently cross-checked 56 candidate records using the available source material, confirmed the screening decisions for the remaining 229 records, and checked the treatment-exposure descriptions for the five studies in **Table 1**. **Supplementary Table S1** records the verification scope for each PMID. This sequential screening and checking process did not involve independent duplicate screening. The review remains a targeted narrative synthesis, and the database search was not updated during revision. Single-database coverage, retrospective eligibility criteria, the absence of duplicate independent screening, and incomplete source access remain potential sources of selection and extraction bias.

## 2 Nephrin as an autoantigen: structural and signaling vulnerability

### 2.1 Extracellular binding can change intracellular behavior

Nephrin spans the podocyte membrane and contributes to the slit diaphragm between adjacent foot processes. Its extracellular domains support cell contacts, while its intracellular tail connects those contacts to signaling proteins and actin. Khoshnoodi and colleagues demonstrated nephrin self-interaction and nephrin-dependent contacts in transfected cells. Antibodies against its first two extracellular immunoglobulin-like domains disrupted these contacts (3). This experiment gives a direct basis for an adhesion mechanism, although individual patient autoantibodies need not recognize the same domains.

Nephrin also senses events outside the cell. Experimental engagement of its extracellular domain activates Src-family signaling, promotes tyrosine phosphorylation, recruits Nck, and supports actin assembly (4). Nck-dependent connections are required for normal podocyte structure, and inducible loss of Nck in adult mice causes proteinuria and glomerular damage (5, 6). Phosphorylation therefore cannot be treated as a general injury marker. Its effect depends on the signaling context, the sites involved, and the organization of nephrin at the membrane. A useful functional experiment must measure those changes together with barrier function or cell structure. Phosphorylation alone cannot distinguish an adaptive response from the start of injury. This distinction also argues for testing a signaling intervention against normal nephrin function before considering it as a treatment target.

Antibody-driven clustering offers one route from binding to dysfunction. Experimental work shows that multivalent nephrin-Nck interactions set a threshold for clustering and promote nephrin internalization (7). We infer that autoantibody affinity, epitope location, and binding valency could shift this threshold in patients. This model explains why two patient autoantibodies with similar assay signals might have different effects on the filtration barrier. Direct comparisons of patient-derived monoclonal antibodies would test that inference.

### 2.2 Experimental injury reveals a signaling route

Zhang and colleagues studied rats given murine anti-nephrin antibody, isolated rat glomeruli, cultured human podocytes, and podocyte-specific ephrin-B1 knockout mice. Antibody-induced phosphorylation of nephrin and ephrin-B1 depended on calcium entry through transient receptor potential canonical channel 6 (TRPC6). Ephrin-B1 then released the polarity protein Par6, which stabilized and activated Cdc42. Increased Cdc42 activity engaged calcineurin-Snail signaling, reduced nephrin and ephrin-B1 expression, and promoted claudin-1 expression through the transcription factor Stat3 (8). These experiments connect extracellular antibody binding to loss of the specialized podocyte phenotype. They establish a pathway in an experimental antibody model; studies using patient autoantibodies can now determine how often this pathway operates in human disease.

Antibody class and glycosylation add further variation. Shu and colleagues detected both IgG and immunoglobulin M (IgM) anti-nephrin responses in adults with MCD or primary FSGS (9). Guo and colleagues reported IgG4 predominance and a type 2 cytokine profile in an MCD cohort (10). Spinelli and colleagues found reduced IgG fucosylation in patients who tested positive for anti-nephrin autoantibodies. Their lectin assay measured overall IgG glycosylation (11). These findings identify antibody class and glycosylation as candidate modifiers of injury; testing the nephrin-specific fraction would determine whether it shares the overall IgG glycosylation pattern. A separate study showed that poorly sialylated patient IgM disrupted cultured podocytes and that resialylation restored cellular features toward control conditions; that experiment examined IgM glycosylation without establishing nephrin-specific targeting (12). Antigen recognition and antibody-mediated injury can therefore converge through more than one route.

## 3 Pathogenic evidence: from human recognition to experimental injury

### 3.1 Human serum and tissue identify a coherent target response

The original NEPTUNE analysis detected anti-nephrin autoantibodies in 18 of 62 patients with biopsy-proven MCD. Eleven positive patients with samples obtained in complete remission became seronegative. Their active-disease sera also immunoprecipitated nephrin from human glomerular extracts. In a separate tissue-selected cohort, all nine patients with punctate podocyte IgG and available active-disease serum were positive, whereas 12 controls were negative (1). These linked observations support target recognition at the site of injury and a relationship with disease activity. The tissue-selected comparison establishes concordance in that setting; it does not provide an unselected diagnostic accuracy estimate.

The larger multicenter study detected autoantibodies in 46 of 105 adults with MCD, 7 of 74 with primary FSGS, and 94 of 182 children with idiopathic nephrotic syndrome. Detection reached 69% in untreated adults with active MCD and 90% in untreated children with active disease (2). These findings show that sampling state strongly affects the observed frequency. They also show why childhood idiopathic nephrotic syndrome must retain its clinical label when biopsy confirmation is absent.

### 3.2 Experimental models establish injury-producing capacity

Experimental anti-nephrin antibodies caused proteinuria before the human autoantibody discovery. Topham and colleagues identified rat nephrin as the target of the nephritogenic monoclonal antibody 5-1-6 (13). Takeuchi and colleagues subsequently induced nephrotic syndrome, podocyte loss, and FSGS lesions in C57BL/6 mice using polyclonal rabbit anti-mouse nephrin antibody (14). These studies establish that antibody targeting of nephrin can disrupt the filtration barrier and can produce different structural outcomes.

Hengel and colleagues moved this argument into an autoimmune setting by immunizing mice with recombinant murine nephrin. The mice developed nephrotic syndrome, slit diaphragm IgG, nephrin phosphorylation, and podocyte changes resembling MCD (2). Active immunization demonstrates that an immune response directed against nephrin can generate disease. The model includes the host immune response and differs from transfer of a defined antibody preparation.

Investigators transferred total IgG from a patient with anti-nephrin autoantibody-positive MCD into a rabbit after demonstrating that the patient’s autoantibodies recognized rabbit nephrin. The study used one treated rabbit and one control. The treated rabbit developed proteinuria and foot process effacement. Glomerular IgG recovered from its kidney recognized nephrin, and the tissue showed increased nephrin phosphorylation (15). This experiment demonstrates transferable injury from the patient’s total IgG with target engagement in the recipient kidney. It did not isolate the nephrin-specific fraction or establish its sufficiency for injury.

The recipient kidney lacked the electron-dense deposits expected in a conventional deposit-forming glomerulopathy. This result fits a model in which antibodies disturb a surface junction and its signaling machinery (15). The available experiments therefore connect nephrin targeting to protein leak through both structural and signaling effects. They also show why species recognition must be established before a negative transfer experiment is interpreted: an antibody cannot test the proposed mechanism in a recipient whose nephrin it fails to bind.

Target recognition, temporal association, functional injury, and antigen-specific attribution answer distinct causal questions. Human serology and tissue findings establish target recognition and clinical concordance, while experimental antibodies and immunization establish injury-producing capacity. Transfer of patient total IgG extends that capacity to a human preparation, with the active fraction still unresolved. Depletion followed by add-back would test dependence on nephrin-reactive IgG. Purified patient autoantibodies or derived monoclonal antibodies could establish sufficiency under defined conditions; studies with independent donors and models would test generalizability.

### 3.3 The source of recurrent autoantibody production remains a separate question

In a steroid-treatment study, autoantibody loss and clinical remission accompanied changes in circulating B-cell populations (16). These observations connect treatment, serology, and the immune compartment, but bulk plasmablast measurements do not identify nephrin-specific clones. Reports involving impaired FOXP3-dependent tolerance or immune checkpoint inhibition also connect disturbed immune control to nephrin-associated disease (17). Antigen-specific B-cell and plasma-cell studies should determine which cells sustain the response and which survive remission. **Table 2** summarizes the studies that anchor the pathogenic and clinical arguments. **Figure 1** integrates the mechanistic pathways and complementary pathogenic evidence.

**Table 2:** Original evidence for nephrin-directed podocyte injury.

Study	Population or model	Main result	Interpretation
Watts et al., 2022 (1)	62 biopsy-proven MCD; separate tissue-selected cohort.	18/62 positive; all 11 paired positive cases became negative in complete remission; all 9 tissue-selected IgG-positive cases with serum were seropositive.	Serum/tissue targeting and activity; selected tissue cases cannot estimate diagnostic accuracy.
Hengel et al., 2024 (2)	539 patients, 117 controls; mouse nephrin immunization.	Positive: adult MCD 46/105, primary FSGS 7/74, children 94/182. Immunized mice developed nephrotic disease and slit diaphragm changes.	Human associations and autoimmune injury in mice; pediatric labels were not all biopsy-defined MCD.
Hengel et al., 2025 (15)	Patient total IgG transfer: 1 treated rabbit, 1 control.	Proteinuria and foot process effacement; recovered glomerular IgG recognized nephrin.	Patient total IgG transferred injury; whether the nephrin-specific fraction is sufficient remains untested.
Zhang et al., 2025 (8)	Antibody-injected rats, rat glomeruli, human podocytes, ephrin-B1-deficient mice.	TRPC6-dependent Par6 release and Cdc42 activation altered nephrin, ephrin-B1 and claudin-1 expression.	Functional injury pathway established with experimental anti-nephrin antibody.
Shu et al., 2025 (9)	596 adults: MCD 436; primary FSGS 160.	IgG/IgM positivity: either 43%, IgG 30%, IgM 26%, both 13.1%. Autoantibodies tracked activity and could reappear before relapse.	Class-specific phenotypes and a relapse signal; combined-class rates differ from IgG-only rates.
Liu et al., 2025 (18)	169 patients/controls; multiple antigens and assay platforms.	Two FSGS samples showed strong reactivity; about half of an additional MCD group had weak IP signals missed by on-bead ELISA.	Antigen/platform effects; confirmed positives and unresolved weak signals.
Chen et al., 2025 (19)	108 glomerular-disease cases (36 MCD); 20 healthy controls.	Cell-based assay: 7/36 MCD positive, including 7/26 with nephrotic syndrome.	Complementary cellular detection; limited sensitivity for excluding MCD.
Hengel et al., 2025 (20)	333 children stratified by steroid response/genetics.	Positive: SSNS 69/101; SDNS 19/67; nongenetic SRNS 14/103; genetic SRNS 1/62.	Immune-responsive phenotypes; treatment was not randomized by autoantibody status.
Bianchi et al., 2025 (21)	69 children: SSNS 50, SRNS 15, other genetic diagnoses 4.	SSNS: 13/20 onset samples and 13/24 relapse samples were autoantibody-positive; onset positivity did not significantly predict 12-month relapse.	Activity association; overlapping sample groups are not independent patient counts.
Pecoraro et al., 2026 (22)	Native disease, recurrence and controls; active/remitted disease.	Mouse-cell antigen: 11/20 healthy positive. Human-cell testing: active SSNS 1/9; all SSNS 1/39; SRNS 1/26; recurrence-history cohort 1/34; healthy 1/20.	Proven α-Gal interference; human-cell testing showed no disease enrichment.
Kopač et al., 2025 (23)	17 children; commercial ELISA. Planned sampling: onset/relapse before corticosteroid initiation, remission, and after corticosteroid withdrawal.	No anti-nephrin autoantibodies detected.	Negative findings limit broad disease coverage. Some relapsing children had ongoing immunosuppression; cross-platform comparability was untested.
Hengel et al., 2026 (16)	26 adults; baseline sera 17; week-8 sera 22.	IP-WB: 12/17 positive; 9 exceeded the quantitative cutoff. All 12 positive patients remitted with autoantibody loss.	Serologic response and within-cohort readout/cutoff discordance.
Batal et al., 2024 (24)	38 transplant recipients: 21 recurrent, 17 nonrecurrent.	Pretransplant autoantibody positivity: 8/21 recurrent versus 0/17 nonrecurrent; sensitivity 38%, observed specificity 100% for predicting diffuse podocytopathy recurrence.	Pre-event risk signal; negativity missed most recurrences.
Shirai et al., 2026 (25)	4 autoantibody-positive recurrent, 2 autoantibody-negative recurrent, 3 nonrecurrent cases.	All 4 positive cases recurred on day 1; IgG localization and nephrin loss appeared 1 hour after reperfusion.	Immediate circulating injury; this series does not estimate prevalence.
Raglianti et al., 2026 (26)	213 cases (tissue and/or serum); 258 controls.	Nephrin-only reactivity tracked minimal-change/steroid-sensitive phenotypes; other-target patterns tracked less favorable courses.	Antigen-related phenotypes; anti-slit diaphragm positivity is broader than anti-nephrin positivity.
Lai et al., 2026 (27)	191 pediatric serum samples; lateral-flow assay versus IP-WB.	92% agreement; nephrin inhibition removed signals in 12 lateral-flow-positive, IP-WB-negative samples.	Rapid target-related detection; samples were not necessarily unique patients.

Studies differ in patient selection, disease activity, treatment exposure, antigen preparation, and positivity thresholds. Detection rates and estimates of diagnostic or prognostic performance therefore cannot be compared directly across studies or used to rank assays. Batal’s sensitivity and observed specificity estimates apply to recurrence prediction in one selected transplant cohort, not to general analytical assay performance. Rates use each study’s sampling denominator and are not pooled. ELISA, enzyme-linked immunosorbent assay; FSGS, focal segmental glomerulosclerosis; IgG/IgM, immunoglobulin G/M; IP-WB, immunoprecipitation-Western blotting; MCD, minimal change disease; SDNS/SRNS/SSNS, steroid-dependent/resistant/sensitive nephrotic syndrome; TRPC6, transient receptor potential canonical channel 6.

**Figure 1:**
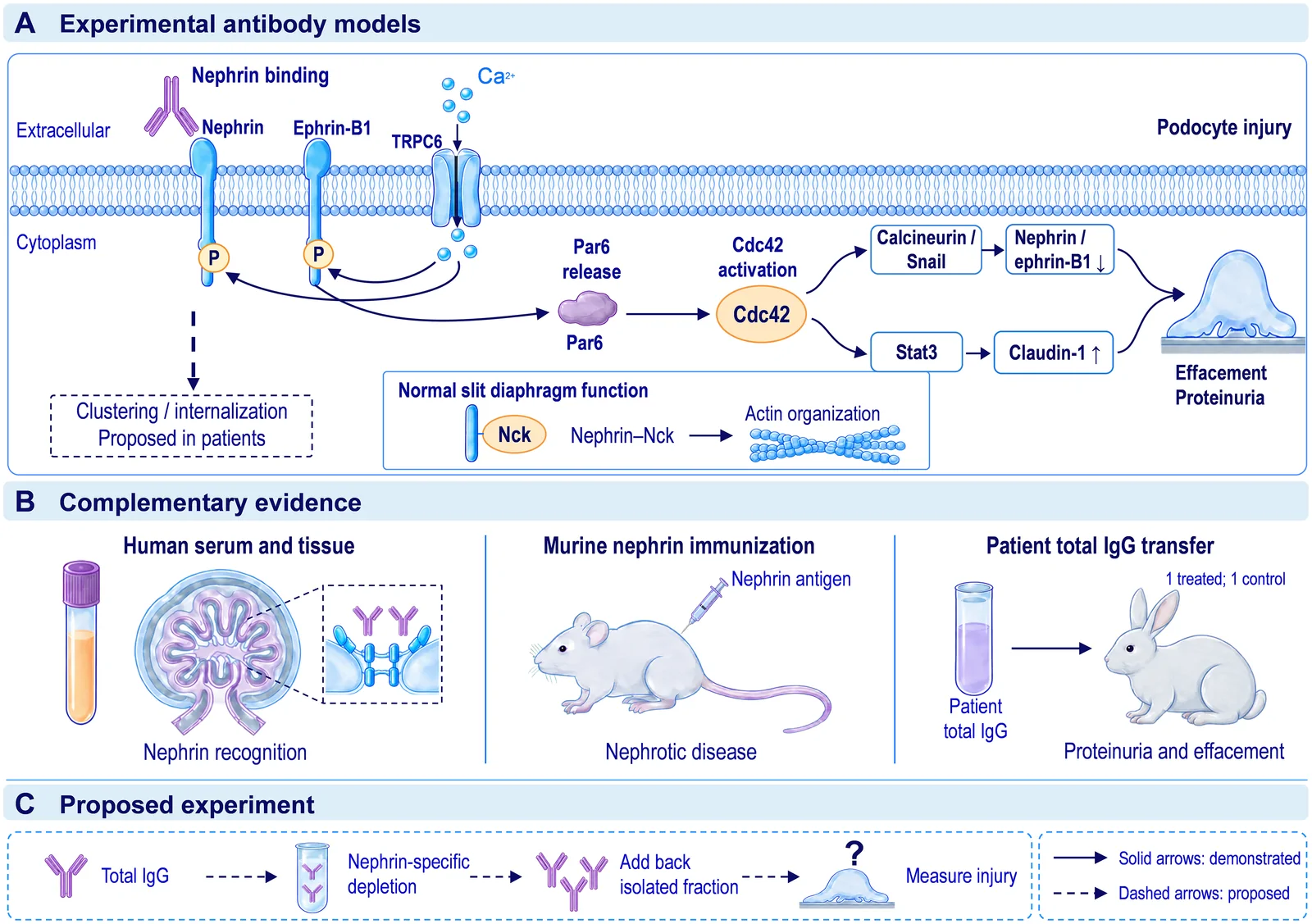
Anti-nephrin autoimmunity: mechanisms and pathogenic evidence. **(A)** Experimental anti-nephrin antibodies activate the TRPC6–ephrin-B1–Par6–Cdc42 pathway and downstream podocyte injury. Normal nephrin–Nck signaling supports actin organization; autoantibody-driven clustering and internalization are proposed in patients. **(B)** Human serum and tissue recognition, murine nephrin immunization, and transfer of patient total IgG provide distinct, complementary evidence. The nephrin-specific fraction was not isolated in the transfer experiment. **(C)** Nephrin-specific depletion and add-back are proposed tests of the pathogenic fraction. Solid arrows indicate demonstrated links within the specified studies or models; dashed arrows indicate proposed links or experiments. IgG, immunoglobulin G; TRPC6, transient receptor potential canonical channel 6. Based on (1, 2, 4–8, 15).

## 4 Assay discordance: analytical variation and disease state

The methodological comparison below draws on the targeted assay-study selection described in Section 1.1. **Table 1** presents five illustrative studies; **Supplementary Table S1** documents the complete record-level screening decisions and study-level extraction.

### 4.1 The antigen preparation is part of what the test measures

An anti-nephrin result depends on the nephrin sequence presented, its folding and glycosylation, the expression cell, purification tags, assay format, secondary antibody, and positivity threshold. Human nephrin produced in mouse cells remains human nephrin at the protein-sequence level. Its host-derived glycans can nevertheless create antibody binding unrelated to the intended protein target.

Pecoraro and colleagues demonstrated this problem directly. With mouse-cell-produced human nephrin, 11 of 20 healthy volunteers tested positive. The recombinant antigen carried α-Gal, and α-Gal absorption or enzymatic removal reduced the misleading reactivity. Human-cell-produced nephrin removed this interference (22). These experiments establish glycan recognition as a source of false attribution to nephrin. Competition with the same glycosylated preparation can retain that error because the competing material carries the unwanted epitope. This explanation applies to the affected antigen preparations. Both Watts and Hengel produced their human nephrin antigens in human embryonic kidney 293 (HEK293) cells, so α-Gal on mouse-cell antigens cannot account for their positive findings (1, 2).

Liu and colleagues also found broad reactivity to a preparation from mouse myeloma NS0 cells. Switching from His-tagged to FLAG-tagged antigen removed signals in selected control samples; a His-peptide blocking experiment confirmed direct anti-tag binding in one of them (18). Their peptide-N-glycosidase F treatment did not abolish background reactivity. That procedure and targeted α-Gal removal tested different aspects of glycan interference. Together, these studies show that the expression host and purification tag can change the apparent target of a serum response.

### 4.2 Platforms recover different parts of the antibody response

The early positive studies already differed in how they defined a positive result. Watts used a plate enzyme-linked immunosorbent assay (ELISA) with HEK293-F-derived, His-tagged nephrin and a threshold of 187 U/mL, the highest value among 30 healthy controls (1). Hengel used HEK293-6E-derived nephrin with His and Twin-Strep tags and classified samples by a specific band after blinded immunoprecipitation-Western blotting. Conventional ELISA did not distinguish their immunoprecipitation-positive samples from negative samples (2). The contrast shows that antigen source alone does not explain platform behavior.

Liu and colleagues compared plate ELISA, immunoprecipitation-Western blotting, an on-bead ELISA, and cellular or tissue-based approaches. Two FSGS samples gave strong, reproducible nephrin recognition. Approximately half of an additional MCD group showed weak immunoprecipitation signals that were not detected by the on-bead ELISA (18). The study therefore supports both robust reactivity in selected patients and a zone of weak, platform-dependent binding.

A plate assay can change antigen orientation or expose normally inaccessible sites. Immunoprecipitation allows binding in solution but depends on capture, washing, and signal interpretation. A cell assay presents nephrin in a membrane setting, although expression, processing, and accessibility can differ from those in a mature podocyte. Using a cell-based assay, Chen and colleagues detected autoantibodies in 7 of 36 adults with MCD. Their reported specificity was high within the selected comparison population, but the low detection rate limits its use as an exclusion test (19).

The 2026 steroid study demonstrates that classification within one cohort depends on the readout and cutoff. Immunoprecipitation-Western blotting identified 12 positive baseline samples among 17 available, whereas nine exceeded the quantitative immunoprecipitation-ELISA cutoff of 25 RU/mL. The other three positive samples measured 5, 10, and 24 RU/mL (16). Their classification changed with the readout, independently of population differences. Calibration also differed across studies: Watts used a positive patient serum, Liu used a commercial anti-nephrin antibody, and Hengel quantified recovered nephrin against a recombinant-protein standard (1, 2, 18). These numerical scales describe their own assay systems. A value in one system cannot be read against another system’s cutoff.

New formats may improve access. Lai and colleagues evaluated a quantum-dot fluorescent lateral-flow assay in 191 pediatric serum samples and reported 92% overall agreement with immunoprecipitation-Western blotting. Nephrin inhibition removed the signal in 12 lateral-flow-positive, immunoprecipitation-negative samples (27). This result supports target-related binding in discordant specimens and argues against treating the comparator as infallible. Agreement between platforms still requires clinical validation in the population where the rapid test will be used. **Figure 2** summarizes the sources of assay discordance and the shared-sample comparison needed to resolve them.

**Figure 2:**
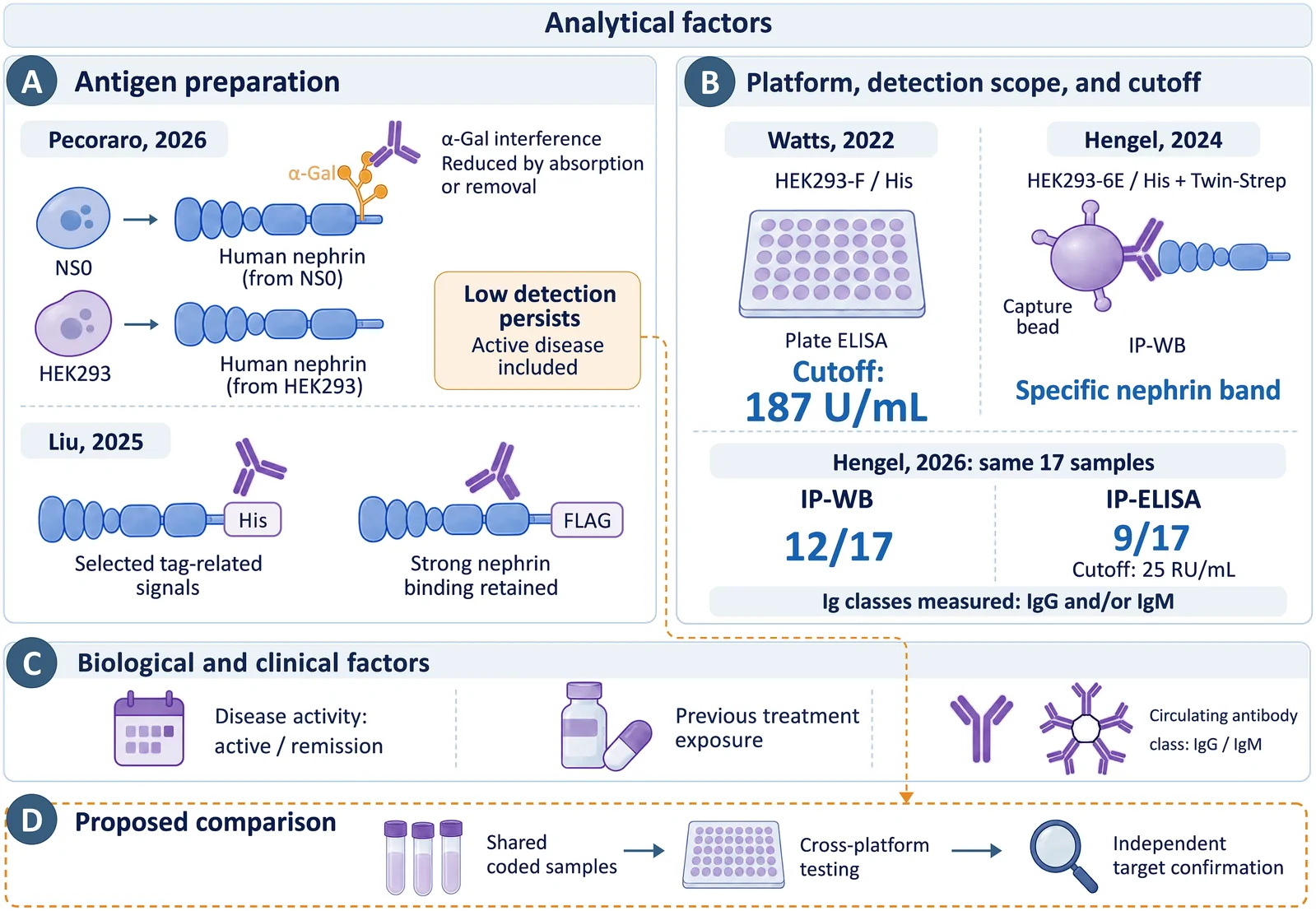
Sources of discordance in anti-nephrin autoantibody detection. **(A, B)** Analytical factors include antigen source and glycosylation, purification tags, platform, immunoglobulin classes measured, and positivity thresholds. The same 17 pretreatment samples illustrate platform-dependent classification. **(C)** Biological and clinical factors include disease activity, previous treatment exposure, and circulating antibody class. The antibody classes measured by an assay and those present in a patient are distinct considerations. Low detection with human-cell-derived antigen remains unresolved. **(D)** Shared coded specimens with independent target confirmation permit assessment of analytical discordance; clinically characterized cohorts are also needed to assess patient-related variation. ELISA, enzyme-linked immunosorbent assay; IP, immunoprecipitation; WB, Western blotting; IgG/IgM, immunoglobulin G/M; RU, relative units. Based on (1, 2, 9, 16, 18, 22).

### 4.3 Treatment and case selection explain part of the variation

Sampling adds a separate source of variation. In the Watts cohort, 97% of patients had started treatment before their earliest available serum sample was collected; 18 of 62 were autoantibody-positive (1). Hengel found autoantibodies in 44% of the overall adult MCD group and 69% of the untreated active subgroup (2). Shu measured both IgG and IgM: either-class positivity was 43% across 596 adults and 51.1% among untreated patients with nephrotic-range proteinuria (9). These comparisons make disease state, treatment, and isotype definition essential to the denominator. Shared specimens can quantify analytical differences, while clinically characterized cohorts are needed to assess disease state and treatment effects.

Clinical context does not resolve all negative findings. In the Pecoraro cohort, 30 of 39 steroid-sensitive patients were in remission. Human-cell antigen testing detected autoantibodies in 1 of 9 active steroid-sensitive cases and none of 30 in remission, yielding 1 of 39 overall (2.6%). Positivity was also low in patients with steroid-resistant disease (1/26) and those with a history of transplant recurrence (1/34). One of 20 healthy volunteers was positive, and tissue studies did not show the expected IgG-nephrin pattern (22). Kopač and colleagues reported no detectable anti-nephrin autoantibodies with a commercial ELISA in a study of 17 children. Sampling was scheduled before corticosteroid initiation at onset or relapse, at remission, and after corticosteroid withdrawal; some relapsing children were nevertheless receiving corticosteroids or other immunosuppressants. The negative findings cannot simply be attributed to sampling after corticosteroid initiation, although prior or ongoing treatment effects were not excluded. Comparability with assays used in positive studies was not tested (23). These low or absent signals limit claims of broad disease coverage; analytical validity and biological differences require further study.

**Table 1** compares antigen preparations, positivity rules, sampling conditions, and confirmatory findings of key assay studies. Glycan interference, selected tag-related false positives, and platform-dependent classification explain documented analytical differences. Residual discordance may also reflect genuine differences in endotype frequency, other disease mechanisms, or binding without pathogenic activity. Positive and negative cohorts require the same scrutiny of selection, treatment, thresholds, and target confirmation. Testing shared coded specimens quantifies platform differences while controlling for patient variation; clinically characterized cohorts are also needed. Until those comparisons resolve the differences, current evidence supports a specific molecular endotype with uncertain prevalence across MCD and FSGS.

## 5 From autoantibody reactivity to disease endotypes

### 5.1 Adult and pediatric cohorts define overlapping immune phenotypes

Shu and colleagues studied 436 Chinese adults with MCD and 160 with primary FSGS. They detected anti-nephrin IgG in 30%, IgM in 26%, and either class in 43%; dual positivity occurred in 13.1%. Positive patients had more severe nephrotic features and a less favorable relapse course (9). These data support an autoantibody-associated phenotype across both pathological categories. The combined IgG-or-IgM rate should not be compared directly with IgG-only studies.

In Hengel’s adult cohort, anti-nephrin autoantibodies were detected in 46 of 105 patients with MCD (44%) and 7 of 74 with primary FSGS (9%) (2). These findings identify a smaller detectable autoantibody-positive subset in primary FSGS and limit generalization of this mechanism to FSGS as a whole. Seronegativity alone does not establish the absence of nephrin-autoantibody-mediated injury. Within the autoantibody-positive subset, MCD and FSGS may differ in the balance between reversible dysfunction and lasting podocyte loss. Transient disruption of slit diaphragm organization and signaling could permit recovery, whereas sustained injury and podocyte depletion could favor scarring. Experimental anti-nephrin antibodies can cause podocyte loss and segmental sclerosis (14), but this does not establish a fixed MCD-to-FSGS sequence in humans. Epitope, affinity, binding valency, subclass, and glycosylation are candidate modifiers of injury, as discussed in Section 2. Host repair capacity, pre-existing damage, and the duration of autoantibody exposure may further influence recovery. The cited human cohorts do not establish which factors determine the biopsy phenotype. The available glycosylation study measured overall IgG, so nephrin-specific effects remain unresolved (11). Independent adult studies associate anti-nephrin reactivity with nephrotic severity and steroid dependence (28, 29), but do not distinguish these causal alternatives. Longitudinal studies combining autoantibody characterization, functional assays, and structural outcomes could test these alternatives.

Pediatric studies add treatment and genetic information. In a multicenter cohort, autoantibodies were detected in 69 of 101 children with steroid-sensitive disease, 19 of 67 with steroid-dependent disease, 14 of 103 with nongenetic steroid resistance, and 1 of 62 with genetic steroid resistance. Among active nongenetic steroid-resistant cases, 13 of 74 children responsive to intensified immunosuppression were positive, compared with none of 17 with multidrug resistance (20). These findings identify an immune-responsive subgroup within steroid resistance. They do not make a negative result evidence against all immune mechanisms. Bianchi and colleagues also frequently detected autoantibodies at disease onset and relapse in children, with lower levels during remission (21).

Tissue can sharpen this interpretation. IgG-nephrin colocalization was present in 21 of 26 active immune-mediated cases in one biopsy study (30). Simple IgG dusting is less discriminating: a study of 371 MCD biopsies found punctate staining in both primary and secondary disease (31). Other slit diaphragm targets also matter. Raglianti and colleagues linked anti-slit diaphragm staining to treatment response, then identified nephrin, podocin, and KIRREL1-associated patterns with different clinical courses (26, 32). Anti-slit diaphragm positivity therefore cannot be assigned wholesale to nephrin.

We propose a research interpretation that combines serum specificity, tissue targeting, and functional evidence. Concordant findings strengthen mechanistic attribution; isolated low-level reactivity needs further characterization. A negative result may reflect absence of this response, treatment-associated autoantibody loss, limited assay detection, or a different disease mechanism. This approach defines candidate endotypes for investigation, without creating a clinical diagnostic score.

### 5.2 Transplantation provides a temporal test

Nephrin-deficient children who develop anti-nephrin alloantibodies after receiving a nephrin-expressing graft provide an early human example of target-directed injury. Their response follows exposure to a newly expressed graft antigen and differs immunologically from autoimmunity in a native kidney (33). In nongenetic disease, Shirai and colleagues reported an association between anti-nephrin autoantibodies and pediatric post-transplant FSGS recurrence (34). Batal and colleagues then tested pretransplant serum: 8 of 21 patients who later developed recurrent diffuse podocytopathy were positive, compared with 0 of 17 without recurrence. For predicting recurrence, pretransplant seropositivity yielded 38% sensitivity and 100% observed specificity in this selected cohort; these are cohort-specific prognostic estimates, not general analytical properties of the assay (24).

Early graft biopsies add a biological timescale. In four autoantibody-positive recurrent cases, nephrin loss and IgG localization appeared within one hour of reperfusion, accompanied by changes in slit diaphragm partners (25). This timing strongly supports a circulating attack on the graft. The low recurrence-associated detection rate in the Pecoraro study remains a conflicting result (22). Together, the studies justify prospective study of an autoantibody-defined high-risk subgroup, while the limited sensitivity makes seronegativity unsuitable for ruling out recurrence.

## 6 Potential clinical applications and validation needs

### 6.1 Etiologic assessment and disease activity

A reproducible, target-confirmed positive result provides evidence of nephrin-directed autoimmunity in a compatible podocytopathy. Its proposed role in etiologic assessment remains investigational because assay standardization and performance across the relevant differential diagnosis are unresolved. Studies should evaluate its added information alongside biopsy, genetics, disease activity, and treatment exposure. A positive binding result alone does not establish that the autoantibody caused the lesion.

This etiologic interpretation also helps explain selected overlapping phenotypes. In adults with IgA nephropathy and nephrotic-range proteinuria, Si and colleagues found greater autoantibody enrichment in the subgroup with overlapping MCD features (35). The finding supports an additional podocyte-directed process in that subgroup and helps interpret the autoantibody result in the context of the biopsy phenotype.

Serial measurements show consistent associations with disease activity in patients with detectable autoantibodies. Levels fall with remission across several cohorts (1, 2, 9, 10). In the steroid study, 26 adults had analyzable data, and 17 contributed pretreatment serum. All 12 patients who were immunoprecipitation-positive at baseline achieved clinical remission with autoantibody disappearance after eight weeks (16). Serial testing on the same platform therefore characterizes a measurable serologic response. Its added clinical value beyond proteinuria and serum albumin requires prospective evaluation.

A reported transplant case illustrates how serologic and clinical measurements can diverge. Circulating autoantibodies became negative while proteinuria and tissue IgG persisted. The patient later received a second transplant without early recurrence when serum and tissue testing were negative (36). This course supports different timelines for circulating autoantibody clearance, tissue autoantibody persistence, and structural recovery. Prospective follow-up is needed to determine whether these differences distinguish ongoing immune injury from residual damage. **Figure 3** summarizes potential clinical applications and the trials needed to test treatment decisions.

**Figure 3:**
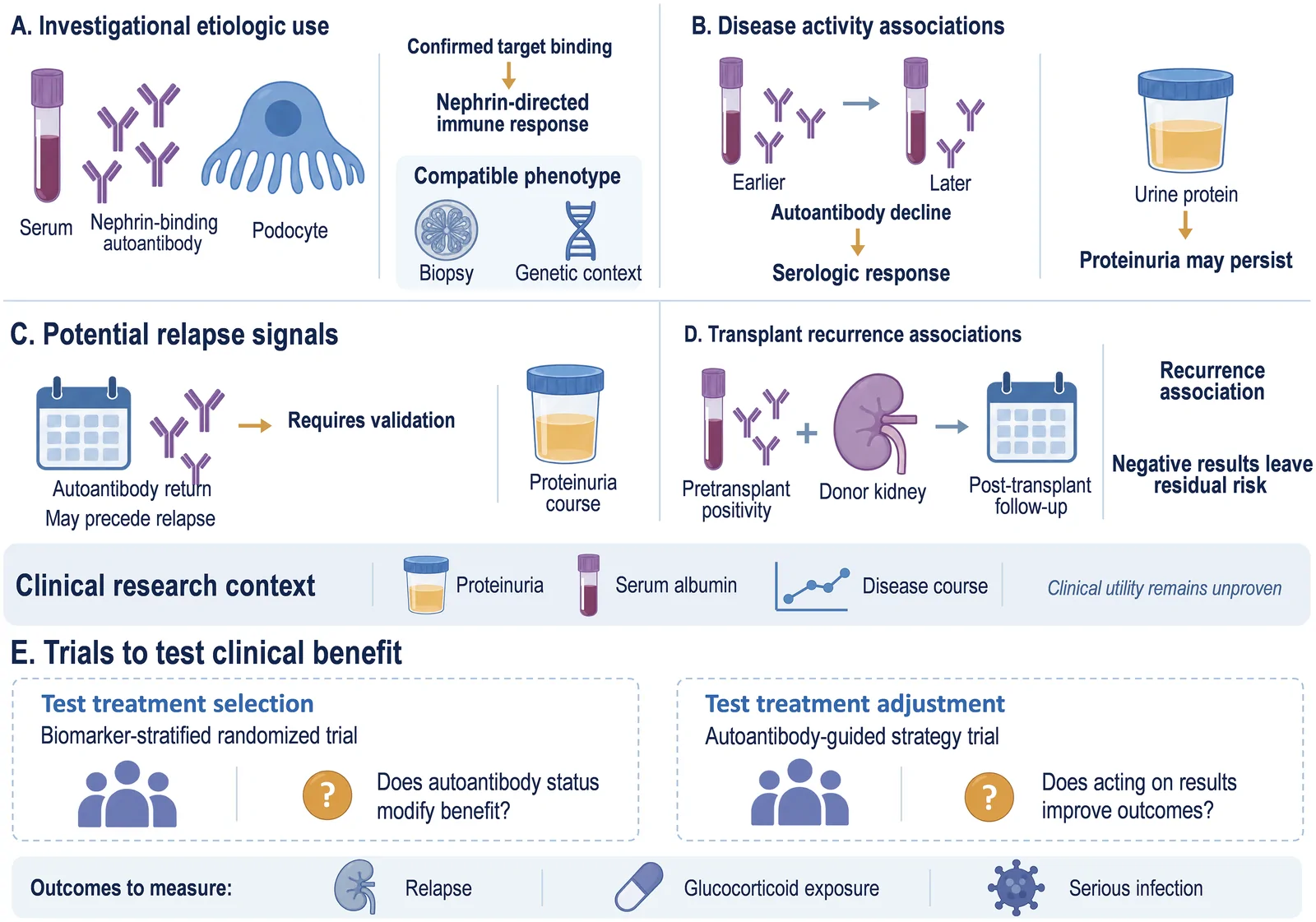
Potential clinical applications of anti-nephrin autoantibody testing. **(A)** Confirmed target binding supports studies of etiologic characterization within the biopsy and genetic context. **(B)** Serial autoantibody decline reflects a serologic response, while proteinuria may persist. **(C)** Autoantibody reappearance has preceded relapse in some patients; its predictive value requires validation. **(D)** Pretransplant positivity is associated with recurrence in selected cohorts, and negative results leave residual risk. These observational findings support investigation of potential applications. Assays remain unstandardized, and clinical benefit from autoantibody-guided management has not been established. **(E)** Biomarker-stratified and autoantibody-guided strategy trials test treatment selection and adjustment using outcomes such as relapse, glucocorticoid exposure, and serious infection. Based on (1, 2, 9, 16, 24, 36–39).

### 6.2 Relapse and transplant recurrence prediction

Shu and colleagues observed anti-nephrin autoantibody reappearance before proteinuria relapse, providing a temporal warning signal in some patients (9). This finding supports prospective testing of autoantibody trajectories alongside urine protein, serum albumin, symptoms, and treatment exposure. The useful warning interval and added predictive value remain to be established. A single positive sample at presentation addresses a different question.

Watts and colleagues observed a shorter relapse-free interval in autoantibody-positive patients, but the comparison did not reach statistical significance. Bianchi and colleagues likewise found no statistically significant difference in 12-month relapse by onset autoantibody status (1, 21). Baseline positivity and serial reappearance address different prediction questions. The predictive value of serial change therefore needs to be evaluated separately from baseline serostatus.

Pretransplant positivity identifies a cohort-specific risk signal that precedes graft exposure (24). In the Batal cohort, autoantibodies were detected in 8 of 21 patients who recurred, so seronegativity leaves substantial recurrence risk. Habbig and colleagues reported sustained autoantibody reduction after rituximab and plasma exchange, followed by more than one year without albuminuria after transplantation in an adolescent. The autoantibodies also recognized NEPH3 (37). This case documents an autoantibody-informed approach with a favorable outcome and provides a strategy for comparative testing. Its benefit over standard transplant planning remains untested.

### 6.3 Treatment selection and current translation

B-cell therapy has proven clinical benefit in an appropriate relapsing population. In a randomized adult trial, rituximab produced a 49-week relapse-free rate of 87.4%, compared with 38.0% with placebo; the hazard ratio for relapse was 0.16 (38). This result supports rituximab for the studied clinical phenotype. Treatment selection can draw on that efficacy evidence, the patient’s previous responses, relapse burden, and treatment toxicity. In autoantibody-positive disease, serial serology provides a research measure of the targeted immune response.

Autoantibody decline after treatment documents a serologic response without establishing which therapy provides greater benefit. The studies reviewed do not show that anti-nephrin-guided management improves outcomes compared with standard management. Treatment selection requires evidence of a treatment-by-autoantibody interaction. Escalation or withdrawal based on serial results requires a strategy trial evaluating disease control and treatment burden. Section 7 outlines these distinct tests of clinical utility.

The PIONEER phase 2 study includes an anti-nephrin autoantibody-positive nephrotic syndrome cohort receiving atacicept. The registry describes an open-label, single-group design and had no posted results at the search cutoff (39). This study uses autoantibody-defined enrollment in therapeutic research. **Table 3** connects each potential application to the available evidence and the next decision to be tested.

**Table 3:** Potential clinical applications and validation needs for anti-nephrin autoantibody testing.

Potential application	Key evidence	Evidence-supported interpretation	Next test
Etiologic assessment	Confirmed serum binding and tissue localization support a nephrin-directed immune endotype in selected podocytopathies (1, 2).	Supports antigen-specific characterization in research cohorts. Added diagnostic value beyond biopsy, genetics and sampling state requires validation.	Consecutive diagnostic cohorts: added accuracy across the relevant differential diagnosis.
Activity monitoring	All 12 baseline IP-WB-positive patients remitted with autoantibody loss at 8 weeks. Serum clearance and proteinuria recovery can separate (16, 36).	Serial decline reflects a measurable serologic response in previously positive patients. Its added value beyond proteinuria and albumin has not been established.	Scheduled sampling: distinguish continuing immune activity from residual injury.
Native-disease relapse	Autoantibodies could reappear before proteinuria relapse. Baseline positivity did not significantly predict relapse in some cohorts (1, 9, 21).	Reappearance may provide an early signal; warning time and added predictive value require validation. Baseline positivity addresses a different question.	Prospective cohorts: quantify warning time and validate added prediction.
Transplant recurrence	Pretransplant positivity: 8/21 recurrent versus 0/17 nonrecurrent cases. An autoantibody-informed treatment case had a favorable outcome (24, 37).	Positivity is a cohort-specific risk signal. Seronegativity cannot exclude recurrence; the benefit of changing care based on the result remains unproven.	Independent absolute-risk estimates; comparative trials of recurrence prevention.
Treatment selection or adjustment	Rituximab reduced relapse in an adult randomized trial. PIONEER includes autoantibody-positive patients receiving atacicept (38, 39).	Rituximab benefit applies to the clinical phenotype studied. These studies do not establish an autoantibody-based rule for choosing or adjusting therapy.	Randomized tests of treatment-by-autoantibody interaction and autoantibody-guided treatment escalation or withdrawal.

This table summarizes investigational applications and the evidence needed for validation. Observed associations and serologic changes do not establish benefit from acting on test results. Assays are not yet standardized, and autoantibody-guided management has not been shown to improve outcomes over standard care. IP-WB, immunoprecipitation-Western blotting.

## 7 Discussion: priorities for mechanistic and clinical translation

### 7.1 Laboratories should compare the same samples

A shared serum panel should include untreated active MCD, primary FSGS, paired remission samples, genetic and secondary disease, autoimmune controls, and healthy volunteers. Samples with established α-Gal or tag-related reactivity would test interference directly. Laboratories should receive coded aliquots and report the antigen sequence, expression host, tag, lot, assay conditions, and positivity rule.

The comparison should measure reproducibility and resolve discordant specimens using alternative antigens, binding formats, and tissue recognition. Investigators should retain quantitative signals, define weak results explicitly, and fix thresholds before external testing. The same specimens can then enter functional studies, linking robust binding to injury-producing capacity.

Antigen-specific depletion and add-back provide the decisive test of which fraction of patient IgG causes injury. Patient-derived monoclonal antibodies can define the relevant epitopes, binding strength, and effector properties. Parallel antigen-specific B-cell studies can identify the cells that sustain or restore the response after remission.

### 7.2 Prospective cohorts should define the timing and added value

Consecutive cohorts should collect samples at presentation and at scheduled follow-up visits, with treatment exposure recorded at each time point. Investigators should define the relapse prediction window before analysis and compare autoantibody trajectories with relapse history, proteinuria, serum albumin, and kidney function. They should measure the warning interval after autoantibody reappearance and test risk estimates in an independent population.

Transplant cohorts should report absolute recurrence risk by pretransplant autoantibody status. Serial native-kidney and transplant studies should also identify patients whose autoantibody clearance and proteinuria recovery diverge. These observations can test whether serology helps distinguish continuing immune activity from residual injury. Total IgG, urinary loss, immunosuppression, and plasma exchange are relevant modifiers of the circulating measurement.

### 7.3 Trials should test the decision that the assay is meant to support

An autoantibody-enriched controlled trial can establish treatment benefit in the selected population. A biomarker-stratified randomized trial can determine whether autoantibody status changes the relative treatment effect. A strategy trial should compare autoantibody-guided escalation or withdrawal with decisions based on standard clinical measures. Each design connects the assay to a defined decision.

The relevant outcomes include relapse, durable remission, cumulative glucocorticoid exposure, serious infection, kidney function, and quality of life. The clinical target is better disease control with less treatment burden. These outcomes allow a direct test of the benefit of autoantibody-guided care.

## 8 Conclusions

Antibody targeting of nephrin is an experimentally established cause of podocyte injury. Human serum and tissue studies support an autoimmune endotype in a subset of patients, and total patient IgG can transfer injury. The nephrin-specific contribution within that preparation and the generalizability of human autoantibody pathogenicity remain unresolved. Establishing those links would connect target recognition to more precise therapeutic studies.

Assay discordance has both analytical and potentially biological explanations. Glycan and tag interference, platform sensitivity, thresholds, treatment, and antibody class affect measured frequencies. Persistently low detection with human-cell antigens also limits generalization across podocytopathies. Shared-sample studies should resolve assay effects while allowing for genuine differences in endotype frequency.

Confirmed anti-nephrin autoantibodies characterize an antigen-specific response, and serial measurements track its circulating signal in positive patients. Autoantibody reappearance and pretransplant positivity show promising temporal associations with risk. Their roles in routine diagnosis, monitoring, prediction, and treatment decisions remain investigational. Standardized assays, prospective validation, and trials comparing autoantibody-guided care with standard management should determine whether these signals improve outcomes.
