# Supplementary material for: Anti-nephrin autoantibodies in podocytopathies: pathogenicity, assay discordance, and translation
Source: Front Immunol. 2026 Oct 26;17:2000932. doi: 10.3389/fimmu.2026.2000932 (PMC13746183; doi:10.3389/fimmu.2026.2000932)
Supplement: Supplementary file 1 [file DataSheet1.docx]

Supplementary Methods S1

Targeted search and revision-stage reassessment of human anti-nephrin assay evidence

This supplement describes the search used for the narrative review and the reassessment, during revision, of human assay studies relevant to Section 4. The full review remains a targeted narrative synthesis. The eligibility rules and record-level decisions below were specified and applied during revision on October 8, 2026. They do not represent a prospectively registered protocol or a reconstruction of undocumented historical screening decisions.

# Historical search and record assembly

The saved search files document a PubMed search performed on September 16, 2026, through the NCBI E-utilities ESearch interface. Three title/abstract queries were used. The saved commands specified no language, publication-year, species, article-type or MeSH filters. The retrieval limit was 1,000 records per query, and all returned records were retained. The exact queries are reproduced below. The database search was not rerun or extended to another database during this reassessment. Publicly accessible original articles and supplements were consulted during revision to verify existing records and extraction fields. These checks did not establish a new literature-search cutoff.

## Query 1 main search

(nephrin[Title/Abstract] OR NPHS1[Title/Abstract]) AND (autoantibod*[Title/Abstract] OR autoimmun*[Title/Abstract] OR "anti-nephrin"[Title/Abstract] OR antinephrin[Title/Abstract])

Search date: 2026-09-16. Records returned: 171.

## Query 2 assay search

nephrin[Title/Abstract] AND (antibody[Title/Abstract] OR antibodies[Title/Abstract]) AND (assay*[Title/Abstract] OR ELISA[Title/Abstract] OR immunoprecipitation[Title/Abstract] OR glycosylation[Title/Abstract] OR "alpha-Gal"[Title/Abstract])

Search date: 2026-09-16. Records returned: 58.

## Query 3 mechanism search

nephrin[Title/Abstract] AND (antibody[Title/Abstract] OR antibodies[Title/Abstract] OR Nck[Title/Abstract]) AND (proteinuria[Title/Abstract] OR phosphorylation[Title/Abstract] OR polymerization[Title/Abstract] OR slit[Title/Abstract])

Search date: 2026-09-16. Records returned: 172.

# Search accounting

The three searches returned 171, 58 and 172 records, respectively, giving 401 occurrences and 283 unique PMIDs after deduplication by PMID (118 duplicate occurrences). The searches also specified three seed PMIDs: 16543952, 41191364 and 42440460. PMID 16543952 was already among the query results; the other two added two unique records, bringing the saved assessment corpus to 285 PMIDs. These totals count records, not eligible studies. Historical files listing full-text candidates and bibliography entries were not treated as sequential screening stages because they do not document record-level inclusion or exclusion decisions for those stages.

# Eligibility for the Section 4 assay comparison

Eligible reports provided original human serum or plasma anti-nephrin autoantibody evidence in acquired podocytopathies, including minimal change disease, primary focal segmental glomerulosclerosis, idiopathic nephrotic syndrome, recurrent post-transplant podocytopathy, and overlapping phenotypes such as IgA nephropathy with minimal change features. Relevant disease and healthy comparison groups were retained. Reports had to provide a cohort detection result, a direct comparison of assay formats or thresholds, analytical validation, or data on human-autoantibody specificity, interference or epitope characterization. Primary-data letters were eligible. Positive, negative, weak and discordant findings were assessed using the same criteria. No additional language, publication-year or open-access restrictions were applied to the saved corpus.

# Reports outside this targeted comparison

Reports fell outside the targeted comparison if they were nonprimary reviews, editorials or commentaries without identifiable new human assay data; experimental or protein-expression studies without a human circulating anti-nephrin assay evaluation; tissue-only studies; or studies of isolated diabetes or congenital NPHS1 alloimmunity outside the acquired-podocytopathy population. Selected clinical cases, antibody-selected tissue follow-up series and patient-IgG transfer studies without a cohort detection analysis or direct assay comparison were retained as clinical or mechanistic context. They were not used to estimate assay detection frequency. An earlier preprint was linked to its final peer-reviewed report to avoid double counting. Reports were not excluded because sufficient original text could not be obtained: uncertain eligibility remained indeterminate, and extraction from otherwise eligible reports could be limited to the abstract.

# Revision-stage assessment procedure

# During the re-evaluation conducted on October 8, 2026, PF reviewed the titles and publication types of all 285 retained records, read available abstracts for potentially eligible or unclear records, and consulted accessible original articles and supplementary materials to resolve ambiguities and extract data. Each PMID was assigned a decision outcome, rationale, source of evidence, and missing information status. XY subsequently cross-checked 56 candidate records using the available source material and confirmed the screening decisions for the remaining 229 records. The 56 candidate records are those whose assessment basis in the Screening worksheet is other than "title and publication type". The "Author verification and scope" column distinguishes candidate evidence cross-checks from confirmation of the other screening decisions. XY also checked the treatment-exposure descriptions for the five studies in Table 2.

# Assessment results and unresolved records

Of 285 records, 24 were judged eligible for the targeted assay comparison, 253 were outside its scope, and eight remained indeterminate. The 253 records outside the scope comprised 61 nonprimary reports, 164 reports without a target human circulating assay evaluation, 15 selected clinical/mechanistic case reports or series, six tissue-only reports, six reports outside the target population and one earlier preprint. The eight unresolved PMIDs were 37495741, 39383467, 39383468, 39383469, 39383470, 40118589, 40975532 and 41042235. They remain in the ledger and were not excluded solely because the original text was unavailable. The 24 eligible records represent reports; they do not establish 24 independent patient cohorts. The extraction notes identify analytical extensions and reused comparator cohorts.

# Extraction and interpretation

Where source information allowed these distinctions, the intended extraction unit was report × assay/platform × clinical group × sampling timepoint. Extracted fields included PMID/DOI and year, design and population, whether denominators counted patients or samples, disease activity, treatment exposure and timing, antigen construct/expression system/tag, platform, positivity threshold and calibration, detection result or method comparison, source locator, access limitations and potential overlap. The accompanying workbook contains all 285 screening records and 24 report-level summaries, with 68 assay/group/timepoint entries based on available data. NV means not verified in available source material; NR means not reported in the reviewed available source; NA means not applicable. NV and NR are not zero. Exact denominators were not inferred from an overall treatment cohort, biopsy cohort, composite multiantigen result or sample count. Units, cohorts and overlapping rows were not pooled. Detection proportions were not relabeled as diagnostic sensitivity. No immunosuppression at collection was distinguished from never having received immunosuppression. Source-locator updates and extraction corrections made during the October 9, 2026 consistency revision did not change the screening decisions, eligible-report set, or literature-search cutoff.

# Role of Table 2 and limitations

Table 2 lists five exemplary studies that directly demonstrate differences in antigen preparation, test readings, thresholds, or sampling contexts. All five are among the 24 eligible reports; the complete screening ledger and data extraction records are available for review. Due to differences in study populations, disease activity, prior treatment, antigen preparation, testing platforms, and thresholds, the positive rates and reported diagnostic performance estimates across studies cannot be directly compared. The historical literature search utilized only one database, was based on keywords in titles and abstracts, and the screening criteria were applied retrospectively. The available source materials remain insufficient to determine the eligibility of several reported studies and certain details regarding testing or treatment. PF's reassessment, followed by XY's candidate evidence cross-checks and confirmation of the other screening decisions, did not constitute independent duplicate screening by two human reviewers. Residual data extraction errors may still exist. These limitations result in potential selection and data extraction biases, and it cannot be claimed that this targeted narrative review is comprehensive.
